# Forensic psychiatry in Rwanda

**DOI:** 10.1080/16549716.2018.1509933

**Published:** 2018-08-29

**Authors:** Ariel Eytan, Alfred Ngirababyeyi, Charles Nkubili, Paul Nkubamugisha Mahoro

**Affiliations:** a Medical Direction, Belle-Idée, Geneva University Hospitals, Thônex, Switzerland; b Huye Isange rehabilitation center, University of Rwanda, Butare, Rwanda; c Neuro-Psychiatric Hospital, Caraes Ndera, Kigali, Rwanda; d Department of psychiatry and Psychotherapy, Valais Hospitals, Monthey, Switzerland

**Keywords:** Rwanda, crime, forensic, hospital, psychiatry

## Abstract

Forensic psychiatry has often been neglected in nonwestern countries, including the African continent. Our aim was to assess the practices and needs for improvement in the field of forensic psychiatry in Rwanda. During a one-week visit conducted in October 2017, we interviewed key-informants working at decisional levels in the domains of health, justice and security. Two clinical workshops involving psychiatrists, psychologists and nurses were held in psychiatric facilities, including at Ndera, the main psychiatric hospital of the country. Three axes of development and improvement were identified: First there is a need for a clearer, more coherent and updated legislative framework. Second, the absence of a forensic secured unit, which compromises both quality of care for forensic patients and security of the other patients and staff, should be remediated. Third, the supervision and training in this specialized domain should be provided through international collaborations. Hopefully, Rwanda could become in the next few years a driving force for other African countries in the field of forensic psychiatry.

## Background

Forensic psychology and forensic psychiatry are located at the interface of the sciences of the psyche and the law. The precise definition of forensic psychiatry varies from one country to another, due to different historical developments, mental health systems and legal traditions [1]. This subspecialty of psychiatry broadly deals with clinical expertise applied in the legal context, embracing civil, criminal, correctional or legislative matters, as defined by the American Board of Forensic Psychiatry [2]. It involves the assessment and treatment of those who are both mentally disordered and whose behavior has led, or could lead, to offending [3]. Even in Western countries, it is a relatively new field, which was officially recognized as a subspecialty by the American Board of Medical Specialties in 1992 [4].

Western and non-western forensic psychiatry systems are addressed in the literature, but data from Africa are scarce [5]. When such descriptions are available, they concern almost exclusively the North and South regions of the continent [6]. However, it was observed that the situation of forensic psychiatry in most sub-Saharan and central African countries is extremely preoccupying. Forensic units are often extensions of prisons, with severe lack of resources in the context of outdated colonial or non-existing mental health legislations. As Dr. Frank Njenga, Founder President of Africa Association of Psychiatrists observed in 2006: ‘A visit to many of these institutions leads to despair about the state of human rights and dignity in our continent. Those who are considered lucky are seen by a demoralized, poorly trained, and inadequately paid doctor who passes by the ward once every few weeks, to see only those patients who are most disturbed. For those who are of no trouble, there is no review. It is against this backdrop of great need that the rest of society is served in the field of forensic psychiatry in sub-Saharan Africa’ [7].

The aim of this study was to assess the state of forensic psychiatry in Rwanda and the needs for future improvements.

## Setting

Rwanda is a small country situated in central Africa and belonging to the eastern African community. The historical development of the Rwandan psychiatric system can be schematically divided in four phases: 1. The pre-colonial period, when traditional approaches were the only form of mental health care. 2. The colonial period, with inputs of western psychiatry. 3. The post-independence period, beginning in 1962, with the development of collaborative projects between Rwanda and Belgium, which allowed for the construction of the sole psychiatric hospital of the country. This facility, called Ndera, is run by the Brothers of Charity and has a capacity of 300 beds. 4. The post-genocide period, starting after the 1994 events.

Indeed, in the spring of 1994, Rwandans endured a cataclysmic civil war and genocide. In 100 days, almost one million people were killed, (nearly 20% of the population at the time), and 250 000 women were raped. Murders and mutilations occurred mainly through machete attacks. Millions were displaced and fled to neighboring countries, mainly Congo and Burundi [8]. Studies have reported that 94% of people in Rwanda during the genocide experienced at least one genocide event including witnessing the murder of family members, having their property and homes destroyed, and having their lives threatened [9]. The overall prevalence of post-traumatic stress disorder (PTSD) in a 2008 survey was 26.1% [10]. The same survey indicated that one in five men (20.5%) and one in three women (30.0%) met diagnostic criteria for PTSD 14 years after the genocide. Prevalence of depression was 22.7% in this study, with 17.8% of all respondents meeting diagnostic criteria for both MDE and PTSD. In another study, Bolton et al. [11] observed a 15.5% prevalence of major depression in a rural southern part of Rwanda 5 years after the 1994 genocidal civil war, a rate three times higher than in South Africa (4.9%), according to an epidemiologic survey published in 2009 [12].

Although Rwanda is known for having low crime rates compared to other African countries, homicides, domestic violence and problems related to substance abuse among the youth seem to be increasing [13,14]. According to the Global Burden of Disease Study [15], interpersonal violence ranked nr. 21 on the list of years of life lost (YLLs) in 1990. YYLs quantify premature mortality by weighing younger death more than older deaths. In 2010, interpersonal violence had risen to the 11th position, which represents a 229% increase, from 0.7% to 2.7% of all causes of premature death.

The main focus of the country on judicial and other reconciliation initiatives during the last two decades left little time and resources to devote to the mental health care of the population, and even less to invest in developing forensic psychiatry. These two issues are, however, coming today to the forefront as top priorities. With a population of approximately 12 million inhabitants, the country is currently undergoing a rapid economic development. The Rwandan government has invested heavily in delivery of quality health care, and physical health indicators have improved dramatically during the last decade [16]. However, there is still a scarcity of mental health professionals and most services are concentrated in the urban areas [17].

In Rwanda, traditional perceptions on mental illness are still highly prevalent. It is common that supernatural beliefs are combined with Christian conceptions of demonic possessions and witchcraft. Therefore, people often seek help from traditional healers or churches. Traditional medicine and biomedical approaches to mental health care coexist, but with little dialogue and interaction [18].

## Method

A twofold qualitative approach was used. On the one hand, key-informants working at decisional and governmental levels in the domains of health, justice and security were interviewed. In order to prioritize flexibility and validity through the construction of an egalitarian relationship between the investigators and the interviewees, we opted for unstructured interviews [19]. Our interview guide included themes related to perceived difficulties and needs for improvement in dealing with forensic patients. On the other hand, we organized two clinical workshops involving psychiatrists, psychologists and nurses in psychiatric facilities, notably at Ndera, the main psychiatric hospital situated in the capital city of Kigali, which is in charge of the forensic cases of the country. Both workshops were divided into three parts: first, a general presentation about current knowledge in forensic psychiatry, given by the investigators; second exchanges about clinical cases brought up by the Rwandan mental health professionals; third, a general discussion and synthesis. The contents of these workshops informed the present study. The most significant findings are summarized hereafter.

## Results and discussion

Both interviews and workshops highlighted three domains of concern.

First, the legal framework was judged too vague and insufficiently detailed. In the corresponding articles of the Penal Code, irresponsibility of the accused is assumed in case of ‘Insanity’, or ‘Etat de démence’, respectively in the English and French versions of the law (Article 101). This label does not correspond to a psychiatric diagnosis in contemporary classifications of mental disorders. Moreover, Article 101 specifies that insanity should have been present at the time the offense was committed in order to conclude to irresponsibility. This may be difficult to prove retrospectively, especially in the absence of a specialized psychiatric assessment shortly after the offense. According to Article 102, such a person should be admitted to a neuropsychiatric facility (in practice, to Ndera hospital). However, no mention is made of prosecution or of a legal follow-up of these cases. Therefore, the decision to release such patients from the hospital relies on mental health professionals only. Psychiatrists may fear to fall under Article 507, ‘Failing to assist a person in danger’, should a problem occur in the post-release phase. A revision of the penal code and the promulgation of a mental health law are expected shortly and should contribute to the settlement of these issues.

Second, the organization of services poses a number of challenges. The lack of specialized professionals was often mentioned in the interviews, despite the fact that there are now 10 psychiatrists in activity throughout the country, including two at Ndera hospital. It is complicated for the juridical authority to obtain forensic psychiatric assessments, due to the absence of specialists. The collaboration between the penitentiary and the health system is described as satisfactory, each prison of the country collaborating with a nearby health center and a local district hospital. There is a strong perceived need, expressed by almost all interviewees, for the implementation of a forensic secured unit at Ndera hospital. Such a unit is expected to have a positive impact both for the treatment of offenders and for the protection of vulnerable patients who are now mixed with the former population. Indeed, 20 patients who have committed crimes or serious offenses are currently hospitalized in general psychiatry at Ndera.

Third, the training of medical students and mental health professionals was a recurrent theme during our visit. A forensic psychiatry module is part of the curriculum of the psychiatric training in Rwanda. However, due to lack of qualified teachers, this module is not currently taught. At the time the survey took place, the department of psychiatry at the University of Rwanda was looking for specialists from abroad to take over this module. Supervisions through telemedicine and courses given by foreign professionals were mentioned as potentially very useful.

Rwanda has developed, during the last two decades, several international collaborations in the field of mental health and psychiatry, notably with European academic institutions in Sweden [20], Germany [21] and Switzerland [10]. There is currently an expressed need for expanding these collaborations in order to develop a modern forensic psychiatry system in the country. Rwanda has several assets, including a strong leadership in the health sector, a clear administrative structure and division into provinces and municipalities, and the presence of a university hospital and faculty of medicine. We therefore believe that Rwanda is in a good position to succeed in this task, and represent an example for other African countries.

## Relevant articles of the Rwandan penal code, English version

**Article 101: insanity of the accused**

There shall be no criminal liability when the accused was suffering from insanity during the commission of the offence. However, a person who has voluntarily deprived him/herself of the use of his/her mind during the commission of the offence shall remain criminally liable, even if such deprivation would have not been caused for the purposes of committing an offence.

**Article 102: admission to a neuropsychiatric facility**

A judge may, at the request of the Public Prosecution, order that a person who shows signs of insanity and who poses a threat to public order be admitted to a neuropsychiatric facility.

**Article 570: concealing an offence or failing to assist a person in danger**

Any person who neglects to assist a person in great danger when there is no risk for him/her or others while in a position to do so or to seek assistance shall be liable to the penalties under Paragraph One of this Article.

**Casevignette**

A 23 years old male patient is brought to Ndera hospital. A few hours before admission, he allegedly killed two unknown people, an elderly man and a young boy, with a machete. He was considered insane and brought in by the police.

Records reveal three prior hospitalizations during the last four years, each time for symptoms of agitation, hostile behavior, incoherent speech, disorientation, self-neglect or undressing in public places. The patient is known for abusing street drugs and alcohol. The main diagnosis was substance induced psychotic disorder. Due to the lack of follow up, the treatment of haloperidol was each time interrupted after discharge.

The patient is the third of four children, from a poor, rural, socioeconomic background. During the 1994 civil war, his parents were displaced in Burundi. The patient spent his early childhood in a refugee camp, than came back to Rwanda after the death of his father. Supported by a charity institution, he attended school for approximately three years. He lived for a while with an elderly sister and her husband, but was turned away because he killed, without any apparent reason, several goats and poultry. He then became a street boy. As a teenager, he started abusing drugs, mainly cannabis, sniffed glue and kanyanga. This last substance is an illicit brew containing up to 50% of alcohol. It sometimes contains methanol, thus causing psychiatric symptoms and even deaths. It is estimated that more than 4ʹ000 Rwandan youth are addicted to this product (http://www.newtimes.co.rw/section/read/229104).

Upon admission, he is in poor physical condition, with an infected wound on both feet. The personal hygiene is also poor. He is in anger, refuses to collaborate or communicate. He is injected with haloperidol and chlorpromazine, and i.v. antibiotics are started. Accommodated in a general psychiatry ward dedicated to ‘agitated patients’, he commits, during the first night in hospital, a third homicide by strangulation of the patient sleeping next to him. Despite his obvious dangerousness, he remains in the same hospital. With antipsychotic medication and abstinence of drugs, the clinical evolution is favorable over the next couple of weeks.

## Paper context

The quality of forensic psychiatric care is poor in most African countries. Both limited resources and cultural factors contribute to this situation. In Rwanda, a rapidly developing country, there is a need for reinforcing this subspecialty of psychiatry. Defining a clearer legal framework, implementing a secured forensic unit, improving the coordination between health and prison services and increasing the competencies of professionals are the priorities. These goals should be achieved in collaboration with western academic centers.
